# The Vitamin D Serum Levels in Pregnant Women Affected by COVID-19: A Systematic Review and Meta-Analysis

**DOI:** 10.3390/nu15112588

**Published:** 2023-05-31

**Authors:** Luiza Szarpak, Stepan Feduniw, Michal Pruc, Michal Ciebiera, Basar Cander, Mansur Rahnama-Hezavah, Łukasz Szarpak

**Affiliations:** 1 Research Unit, Polish Society of Disaster Medicine, 05-806 Warsaw, Poland; luizaszarpak@gmail.com (L.S.); m.pruc@ptmk.org (M.P.); 2 Department of Obstetrics, University Hospital Zurich, Frauenklinikstrasse 10, 8091 Zurich, Switzerland; 3 Department of Gynecology, University Hospital Zurich, Frauenklinikstrasse 10, 8091 Zurich, Switzerland; 4 Research Unit, International Academy of Ecology and Medicine, 02091 Kyiv, Ukraine; 5 Second Department of Obstetrics and Gynecology, Centre of Postgraduate Medical Education, 00-189 Warsaw, Poland; michal.ciebiera@gmail.com; 6 Department of Emergency Medicine, Bezmialem Vakif University, Fatih, 34093 Istanbul, Turkey; basarcander@yahoo.com; 7 Chair and Department of Oral Surgery, Medical University of Lublin, 20-093 Lublin, Poland; mansur.rahnama-hezavah@umlub.pl; 8 Henry JN Taub Department of Emergency Medicine, Baylor College of Medicine, Houston, TX 77030, USA; lukasz.szarpak@gmail.com; 9 Research Unit, Maria Sklodowska-Curie Bialystok Oncology Center, 15-027 Bialystok, Poland; 10 Institute of Outcomes Research, Maria Sklodowska-Curie Medical Academy, 00-136 Warsaw, Poland

**Keywords:** vitamin D, COVID-19, SARS-CoV-2, pregnancy

## Abstract

Vitamin D can modulate immune responses, and its deficiency is linked to increased autoimmunity and susceptibility to infection. In the general population, it has been observed that serum vitamin D levels are connected with the risk of COVID-19 and its severity. Our study aims to examine reported findings on the effect of vitamin D serum levels on infection of COVID-19 during pregnancy. PubMed, Web of Science, Embase, and Cochrane Library were searched for relevant studies. Serum vitamin D serum levels in COVID-19-positive and COVID-19-negative pregnant women were 24.61 ± 20.86 ng/mL and 24.12 ± 17.33 ng/mL, respectively. In mild vs. moderate to critical COVID-19 pregnant women, vitamin D serum levels were 16.71 ± 9.04 ng/mL vs. 10.7 ± 9.37 ng/mL and severe vs. non-severe were 13.21 ± 11.47 ng/mL vs. 15.76 ± 10.0 ng/mL. Only one study reported vitamin D serum levels in the placenta of COVID-19-positive pregnant women compared with the control and results varied and amounted to 14.06 ± 0.51 ng/mL vs. 12.45 ± 0.58 ng/mL, respectively. Vitamin D deficiency tends to be common in pregnant women who have COVID-19, and the level of this vitamin has been demonstrated to have a strong correlation with the severity of the illness. As vitamin D serum levels correlate with COVID-19 symptoms and even with its occurrence, appropriate vitamin D supplementation in the prenatal period is suggested.

## 1. Introduction

The global impact of the COVID-19 pandemic has been far-reaching, affecting a significant portion of the global population, and almost 6.9 million people have died because of the severe acute respiratory syndrome coronavirus 2 (SARS-CoV-2) [1,2,3,4,5]. Pregnancy significantly increases the vulnerability of pregnant women to infectious diseases, especially COVID-19 infection. This susceptibility can be attributed to the physiological changes that occur during pregnancy, such as the suppression of the maternal immune system to safeguard the fetus from potential immune reactions until delivery, as well as anatomical adaptations such as the elevation of the diaphragm in response to the expanding uterus, mucosal edema in the respiratory tract, and increased oxygen requirements [6,7]. Existing evidence suggests that COVID-19 infection may elevate the risk of adverse pregnancy outcomes [8,9,10].

COVID-19 significantly affects the human immune system, reaching beyond the infection of pneumocytes to involve immunological cells. This impact is mediated through the renin–angiotensin system (RAS), where the invasion of cells occurs when SARS-CoV-2 spike proteins bind to angiotensin-converting enzyme 2 (ACE-2) receptors located on cell membranes. This action allows the virus to replicate and invade cells, leading to a reduction in the concentration of ACE-2 receptors and disruption of cellular homeostasis. This process is crucial for the further reproduction and spreading of the virus. Additionally, ACE-2 also plays a vital role in protecting against lung injury, emphasizing the impact of its activity on the severity of pulmonary symptoms observed in COVID-19-infected individuals [11,12,13]. Furthermore, it should be noted that the process of placentation depends on the renin–angiotensin system (RAS), and COVID-19 has been shown to have an impact on this crucial process. Women who are infected with SARS-CoV-2 exhibit a higher incidence of fetal growth restrictions and preeclampsia [13].

To regulate the immune response, vitamin D promotes the proliferation of Th2 lymphocytes while suppressing the proliferation of Th1 lymphocytes [14]. Further studies are performed to discover new vitamin D functions, as our understanding of this vitamin remains incomplete [15]. Vitamin D activity is described as a secosteroid, having antifibrotic, antioxidant, immunomodulatory, and anti-inflammatory actions [16]. To control the immunological response, vitamin D stimulates Th2 lymphocyte proliferation while Th1 lymphocyte proliferation is suppressed [17]. Moreover, it modulates the nuclear factor kappa B pathway [18]. Reports also suggest antiviral properties of vitamin D, as it activates antimicrobial substances and impacts the apoptosis and autophagy of infected cells [19,20]. 1,25-dihydroxyvitamin vitamin D [1,25(OH)D], an active metabolite, has the ability to directly influence viral replication or immunological reactions to infections, including the effect on antimicrobial peptides such as cathelicidin [21]. The ability to inhibit infections was especially shown in pulmonary diseases, such as influenza, and its severity was related to a decreased serum level of vitamin D [22]. Recent meta-analyses have demonstrated a positive correlation between vitamin D supplementation and the course of viral infection as well as COVID-19 itself [23,24,25,26].

Ensuring appropriate lifestyle choices and adequate vitamin supplementation during pregnancy are crucial for optimal fetal development. Among the various vitamin deficiencies, vitamin D insufficiency is particularly common, affecting not only high-risk groups in North American and European countries but also populations in the Middle East and Asia due to modern lifestyles [27,28]. Widespread vitamin D deficiency can be attributed to insufficient exposure of the skin to sunlight, which is necessary for cutaneous synthesis of the vitamin. Inadequate dietary intake alone cannot compensate for achieving appropriate serum levels of vitamin D. Insufficient levels of vitamin D during pregnancy can have long-term consequences, including an increased risk of rickets and other metabolic diseases in developing fetuses; hence, it is essential for fetal development [29,30]. Additionally, emerging evidence suggests potential associations between low vitamin D levels during pregnancy and immunodeficiency, oxidative stress, impaired angiogenesis, and suboptimal placental implantation [31]. Furthermore, certain evidence suggests potential links between vitamin D deficiency and gestational diabetes occurrence, cesarean section, or preterm delivery [32,33,34,35]. Infants born to vitamin D-deficient mothers may face complications such as low birth weight, reduced bone mass, bronchiolitis, asthma, type 1 diabetes, and multiple sclerosis [36,37,38,39,40].

Insufficient levels of vitamin D in the bloodstream contribute to cytokine storms, inadequate protection against apoptosis of epithelial cells, and impaired repair mechanisms of epithelial cells. Consequently, these factors expose the lungs to heightened immune dysregulation, which can have fatal consequences. Moreover, lower serum levels of vitamin D are inversely associated with the presence of pro-inflammatory cytokines, including interleukin 1 (IL-1), interleukin 6 (IL-6), and elevated levels of c-reactive protein (CRP). These cytokines have been linked to the severity of COVID-19 and its adverse outcomes [41,42]. A study conducted in Switzerland found that individuals with COVID-19 have significantly lower vitamin D concentrations than those without the infection [43].

Given the significance of the presented findings, it is crucial to investigate the impact of vitamin D serum levels on pregnant women affected by COVID-19. This meta-analysis aims to systematically evaluate and analyze previously reported studies examining the relationship between vitamin D serum levels and the risk of infection and the severity of COVID-19 illness during pregnancy.

## 2. Materials and Methods

### 2.1. Study Design

This meta-analysis adhered to the Preferred Reporting Items for Systematic Reviews and Meta-Analyses (PRISMA) criteria, and the checklist confirming compliance is provided in the Supplementary Materials, Table S1 [44]. Before the start of the investigation, the research protocol was approved by all authors and registered in the PROSPERO register (International Prospective Registry of Systematic Reviews) with the registration number CRD42022385592.

### 2.2. Search Strategy

Two independent reviewers (M.P. and L.S.) assessed potentially eligible publications individually, with any dispute resolved through additional discussion or arbitrated by a third reviewer (S.F.). The search was conducted across multiple databases from January 2020 to March 2023, including PubMed, Web of Science, Embase, and the Cochrane Library. Additionally, a search was performed on Google Scholar as a supplementary electronic database. The combination of the following keywords was used: “vitamin D” OR “cholecalciferol” OR “vitamin D3” AND “pregnancy” OR “pregnant” AND “COVID-19” OR “SARS-CoV-2” OR “severe acute respiratory syndrome coronavirus-2”. The reference lists of the included studies were manually reviewed to identify any potentially relevant papers. To avoid duplication, only the most recent or complete reports from the same authors were included. Furthermore, the reference lists of relevant publications and systematic reviews were examined to identify additional potentially relevant studies. All references were imported into Endnote (version X9), and duplicate entries were removed before using Rayyan, a software screening tool [45].

### 2.3. Inclusion and Exclusion Criteria

Eligible studies were required to meet the following inclusion criteria: a study comparing vitamin D serum levels in pregnant women with COVID-19 to a control group, mild vs. moderate to critical pregnant women, or severe vs. non-severe pregnant women. Studies that met the following exclusion criteria were excluded: (1) research that failed to produce any of the aforementioned outcomes; (2) studies with no comparable group; (3) studies not published in English; and (4) article types such as editorials, conference papers, reviews, and letters to the editor.

### 2.4. Data Extraction and Quality Assessment

Two independent reviewers (M.P. and S.F.) conducted data extraction using a predefined data extraction form prepared by L.S. Any discrepancies or disagreements between the reviewers were resolved through consultations with the third reviewer (L.S.). The extracted information from the eligible publications included the following: study characteristics (first author, publication year, country of origin, study design, and research groups) and data related to pregnant women (number of participants, age, and vitamin D serum levels among the research groups). To assess the methodological quality of the included studies, the Newcastle–Ottawa Quality Scale (NOS) was employed. The NOS evaluates the quality of a study based on three criteria: selection, comparability, and exposure. Each criterion can be awarded a maximum of four, two, and three stars, respectively. Studies with NOS scores of 7 or higher were considered to be of good quality [46].

### 2.5. Statistical Analysis

The statistical analyses were performed using two software programs: Review Manager (version 5.4, Nordic Cochrane Centre, Cochrane Collaboration, Odense, Denmark) and Stata (version 14, StataCorp, College Station, TX, USA). Odds ratios (OR) with 95% confidence intervals (CIs) were used as the effect measure for dichotomous data, while mean differences (MD) with 95% CI were used for continuous data. All statistical tests were two-sided, and the significance threshold was set at *p* < 0.05. We approximated the means and standard deviations by using the method that was presented by Hozo et al., where continuous outcomes were reported as a median, range, and interquartile range in the included studies [47]. The random-effects model was employed for all the analyses. The degree of heterogeneity was assessed using the *I*^2^ statistic, with values of 25% indicating low heterogeneity, values of 25–50% indicating moderate heterogeneity, and values greater than 50% indicating high heterogeneity [48]. Egger’s test, as well as funnel plots, were used to investigate the possibility of bias in the included studies.

## 3. Results

### 3.1. Study Selection and Characteristics

**Figure 1:**
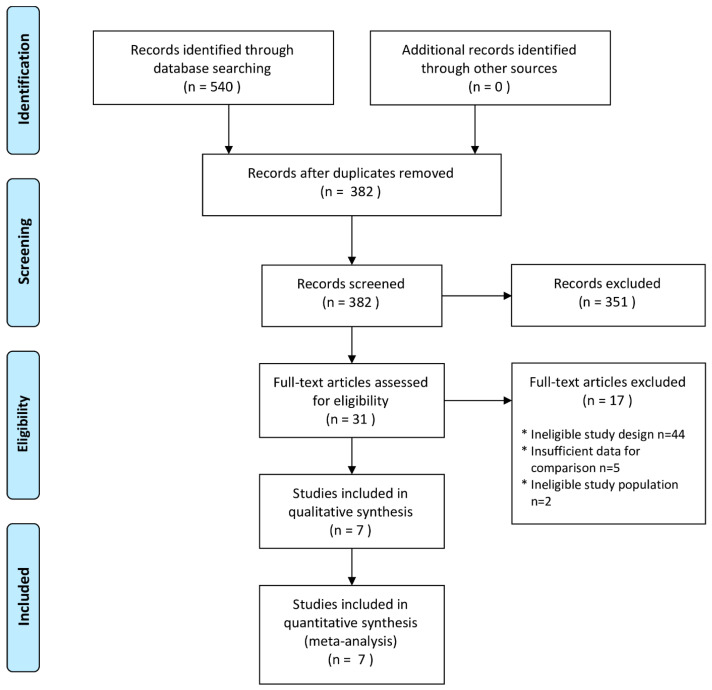
PRISMA systematic review flow diagram.

Figure 1 presents a flow diagram that provides a summary of the detailed study selection process. Initially, a total of 540 articles were identified through the database search. After removing duplicates, 382 publications underwent an initial evaluation based on their titles and abstracts. Just 31 articles were selected for full-text screening based on abstract and title evaluation (Figure 1).

The meta-analysis comprised a total of seven papers [49,50,51,52,53,54,55]. Among these, three studies were conducted in Turkey [53,54,55], two studies in Spain [49,50], one study in Mexico [52], and one in France [51]. The included studies encompassed a total of 1920 pregnant women, 965 of whom were in good health and 955 of whom had been identified to be infected with COVID-19. The included studies encompassed a sample size of 1920 pregnant women, with 965 classified as healthy and 955 identified as COVID-19 infected. Four studies focused on pregnant women in their third trimester [49,50,51,52], whereas the other trials accepted participants of any gestational age. Blood samples were collected in four articles to measure serum 25-hydroxyvitamin D (25(OH)D) levels upon admission [52,53,54,55], two studies obtained samples at the time of delivery [49,50], and one study collected samples within 15 days after a positive RT-PCR COVID-19 test [51]. In all the studies, the optimal serum 25-hydroxyvitamin D (25(OH)D) level was determined to be above 30 ng/mL except for Tekin et al., where values over 50 ng/mL were considered within the normal range for vitamin D [55]. The assay techniques used to detect vitamin D levels varied, with chemiluminescent immunoassay employed in four publications [51,52,54,55]; high-performance liquid chromatography (HPLC) in two papers [49,50]; and one study not specifying the assay method [53]. Six studies compared vitamin D serum levels between COVID-19-positive pregnant women and a control group [49,50,51,52,54,55], three examined mild vs. moderate to critical levels in serum [50,52,54], and three compared severe vs. non-severe COVID-19 cases in pregnant women [50,52,53]. Only one study compared vitamin D serum levels in placental tissue between COVID-19-positive pregnant women and a control group [49]. Detailed characteristics of the included studies can be found in Table 1. Overall, the risk of bias in the included trials was low.

### 3.2. Meta-Analysis

**Figure 2:**
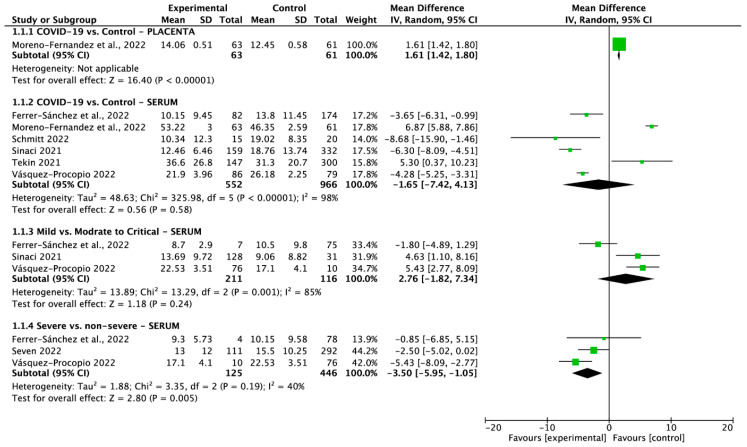
Forest plot of vitamin D serum levels in pregnant women groups. The center of each square represents the standard mean differences (SMD) observed in individual trials, while the accompanying horizontal line represents the 95% confidence interval (CI) [49,50,51,52,53,54,55].

Six studies reported vitamin D serum levels between COVID-19 positive vs. negative pregnant women. Pooled analysis showed that the vitamin D serum levels in serum between those groups were 24.61 ± 20.86 ng/mL vs. 24.12 ± 17.33 ng/mL (MD = −1.65; 95% CI: −7.42 to 4.13; *p* = 0.58; Figure 2). The vitamin D in mild vs. moderate to critical COVID-19 in serum was reported in three studies and was 16.71 ± 9.04 ng/mL vs. 10.7 ± 9.37 ng/mL (MD = 2.76; 95% CI: −1.82 to 7.34; *p* = 0.24; Figure 2). For severe COVID-19 cases compared with non-severe cases, three studies reported values of 13.21 ± 11.47 ng/mL and 15.76 ± 10.0 ng/mL, respectively (MD = −3.50; 95% CI: −5.95 to −1.05; *p* = 0.005; Figure 2). Only one study reported vitamin D serum levels in the placenta of COVID-19-positive pregnant women compared with a control group, with values of 14.06 ± 0.51 ng/mL versus 12.45 ± 0.58 ng/mL (standardized mean difference (SMD) = 1.61; 95% CI: 1.42 to 1.80; *p* < 0.00001; Figure 2).

## 4. Discussion

A comprehensive analysis of risk groups revealed that pregnant women had a 70% higher prevalence of severe COVID-19 infection compared with a similar age group in the general population [7]. Our meta-analysis demonstrated a clear correlation between COVID-19 infection during pregnancy and vitamin D serum levels. Lower levels of vitamin D were observed in pregnant women with more severe symptoms of COVID-19 compared with asymptomatic pregnant women and healthy controls. Several studies conducted by Ferrer-Sánchez et al., Schmitt et al., Sinaci et al., and Vásquez-Procopio et al. consistently showed lower vitamin D serum levels in pregnant women with COVID-19 symptoms compared with healthy pregnant women [50,51,52,54]. However, the studies by Tekin et al. and Moreno-Fernandes et al. reported insignificant results [49,55], suggesting that the combined effect of vitamin D in COVID-19-positive pregnant women was also insignificant.

Ferrer-Sánchez et al., Vásquez-Procopio et al., and Sinaci et al. showed that mild vs. moderate to critical symptoms of COVID-19 have a proportional correlation with higher vitamin D in the serum of pregnant women with increased symptoms of COVID-19 [50,52,54]. Ferrer-Sánchez et al., Vásquez-Procopio et al., and Sven et al. showed decreased vitamin D serum levels in pregnant women with severe COVID-19 symptoms compared with non-severe symptoms [50,52,53].

Moreover, an interesting finding was made by Moreno-Fernandes et al., who showed the increased vitamin D serum level in the placental tissue of COVID-19 pregnant women [49]. To date, no other studies have explored this area. However, this finding suggests a potential direct impact on the fetus, which could contribute to adverse pregnancy and neonatal outcomes. Previous research has established a correlation between low vitamin D serum levels and the severity of placental disorders during pregnancy [31].

As mentioned above, insufficient levels of vitamin D contribute to dysregulated immunological responses, such as cytokine storms, and inadequate protection during COVID-19 infection [41,42]. In COVID-19 patients, cytokine storm leads to increased serum levels of interleukins, particularly IL-8 and IL-10. As reported in Mulchandani et al. study [56], IL-8, as a neutrophil chemotactic factor, stimulates the expression of neutrophil 2 integrin adhesion molecules, while cytokine IL-10 is an anti-inflammatory molecule that works in part to reduce the production and activity of these pro-inflammatory cytokine signals. Increased IL-10 levels can impede proper T-cell responses, leading to T-cell fatigue, polarization of regulatory T cells, and compromised antiviral immune response. Additionally, IL-10 activation induces STAT1, which exhibits both pro-inflammatory interferon (IFN) responses and anti-inflammatory effects on T cells [57]. Consequently, increased levels of IL-8 and IL-10 promote cell activation, oxidative stress, and endothelial damage, which can affect the optimal activation of antiviral T cells. Moreover, both COVID-19-infected and healthy pregnant women exhibit decreased leukocyte levels [58]. These changes contribute to an ineffective antiviral response and the development of severe COVID-19 symptoms [59]. Consequently, pregnant women are particularly susceptible to contracting COVID-19 and experiencing its severe complications.

The RAS regulates trophoblast metabolism and supports placental invasion, circulation, and angiogenesis in normal pregnancies. In healthy pregnancies, there is a relative insensitivity of cells to angiotensin II (ang II), which contributes to maintaining low systemic vascular resistance [60]. During COVID-19 disease, epithelial cells are invaded by the SARS-CoV-2 utilizing a surface spike protein linked to ACE-2 [61]. This, in turn, leads to the synthesis of a complex involving interleukin-1 receptor-associated kinases (IRAK) and toll-like receptors (TLRs), subsequently activating the nuclear factor “kappa-light-chain-enhancer” of activated B-cells (NF-κB) and mitogen-activated protein kinase (MAPK) signaling pathways [62]. Consequently, the humoral response becomes compromised, while T-cells undergo abnormal activation independent of the serum levels of proinflammatory cytokines such as IL-1, IL-6, and tumor necrosis factor-alpha (TNF-α). In severe cases of COVID-19, these cytokine levels are markedly elevated [63,64]. The activation of cytokines and interferon-related responses is also disrupted in this hyperinflammatory state. These dysfunctions give rise to uncontrolled cascades of neutrophil extracellular activity, cellular disturbances, and systemic thrombosis [65]. As a consequence of this hyperinflammation and hypercoagulability, the RAS system becomes hyperactivated.

Vitamin D plays a significant role in COVID-19 infection by modulating various cellular processes. It promotes the downregulation of PI3KC3, Beclin 1, and the mammalian target of rapamycin (mTOR) [66,67], which, in turn, promotes autophagy, lysosomal degradation, and antigen presentation—essential components of the pre-infection defense mechanism [68,69]. Additionally, vitamin D exhibits potential inhibitory effects on renin, an enzyme that induces the expression of angiotensin II (ang II), thereby potentially reducing the severity of COVID-19 [70].

During SARS-CoV-2 infection, the accumulation of Ang II and the reduction in ACE-2 levels contribute to an enhanced respiratory inflammatory response and myocarditis [71]. Vitamin D plays a significant role in regulating the renin–angiotensin system (RAS) by lowering serum renin levels, which, in turn, reduces ACE levels and increases ACE-2 levels. This regulatory mechanism is critical in protecting lung function. Moreover, vitamin D can modulate the synthesis of proinflammatory cytokines while concurrently enhancing the production of anti-inflammatory cytokines. This immunomodulatory effect of vitamin D may have implications for mitigating the cytokine storm associated with the immune response to COVID-19 [72,73]. Studies have shown that T-helper cells (TH1) respond to vitamin D by inducing intrinsic expression of the vitamin D receptor and the enzyme CYP27B1, facilitating both activation and response to vitamin D. In addition, T-helper cells encourage the cells to react to vitamin D by elevating levels of IL-10 while simultaneously lowering levels of interferon-γ. This modulation may help lower or even block the hyperinflammatory response observed in individuals with COVID-19 [74]. Given these mechanisms, it has been suggested that pregnant women with vitamin D deficiency are at an increased risk of severe SARS-CoV-2 infection.

Numerous studies have aimed to determine the optimal vitamin D serum levels during COVID-19 infection. The mean concentration of 25-hydroxy vitamin D [25(OH)D] in the serum, ranging from 30 to 60 ng/mL (75 to 150 nmol/L), has been associated with a reduced risk of severe COVID-19 complications and potential prevention of infection [75,76,77,78]. It has been established that lower levels of vitamin D in the serum are linked to inappropriate immunological responses and may serve as a risk factor for upper respiratory viral infections, including pneumonia [79]. Vitamin D deficiency is one of the most frequent vitamin deficiencies and, notably, it is also one of the most frequent vitamin deficiencies in the population of pregnant women [27,28]. According to Lips et al., severe vitamin D deficiency should be diagnosed when 25(OH)D serum levels fall below 12 ng/mL. The study established a cutoff serum level of 14.5 ng/mL for the occurrence of severe symptoms [80]. To reduce the risk of infection, it is recommended to maintain 25(OH)D serum levels between 40 and 60 ng/mL [81]. However, in nonpregnant women, a comprehensive systematic review and meta-analysis did not establish a clear cause–effect relationship between low serum vitamin D levels (<20 ng/mL) and COVID-19 severity [82].

Vitamin D supplementation could decrease C-reactive protein (CRP) levels and influence leukocyte distribution [83]. Entrenas Castillo et al. showed that high doses of vitamin D3 metabolite (Calcifediol) (administered at a dose of 0.532 mg on the day of admission, 0.266 mg on days 3 and 7, and subsequently once weekly until discharge or admission to the intensive care unit), reduced the need for intensive care unit admission among COVID-19 patients [84]. Unfortunately, there is a lack of studies specifically investigating vitamin D supplementation in pregnant women with COVID-19 infection. The International Endocrine Societies recommend daily supplementation of 5000 IU (up to 10,000 IU in cases of obesity) of 25-hydroxyvitamin D to increase serum vitamin D levels to a minimum of 30 ng/mL [16]. Pregnant women with COVID-19 often present with vitamin D deficiencies and should be prioritized for evaluation of vitamin D deficiency. According to the Eastern European expert consensus statement, deficiency should be addressed with daily supplementation of 6000 IU and an additional 800 to 2000 IU per day of vitamin D3 to prevent deficiency [77]. Supplementation is especially important in winter months; however, the routine evaluation of vitamin D serum levels is not recommended. Yet the Polish guidelines recommend supplementing vitamin D according to vitamin D level [85].

Szarpak et al. conducted a study highlighting the potential reduction in COVID-19 transmission through the implementation of healthy nutritional practices and vitamin D supplementation among vulnerable populations, including pregnant women [25]. This is particularly significant considering the decreased immune response exhibited by pregnant women, making them more susceptible to infections. Despite these promising findings, it is important to acknowledge that further research is warranted to fully comprehend the immunomodulatory and anti-inflammatory properties of vitamin D. In order to mitigate the adverse obstetric outcomes associated with vitamin D deficiency, comprehensive prophylactic programs are imperative, especially during the winter months.

Our analysis encountered several limitations. The number of published articles investigating the correlation between vitamin D and the severity of COVID-19 symptoms in pregnant women was relatively small, and the study groups were not extensive. Moreover, the included articles were published between 2021 and 2022, which is a very short observation period, highlighting the need for further follow-up research to provide more substantial evidence. Another limitation stems from the heterogeneity and retrospective nature of the included publications. Moreover, the scientific literature indicates that vitamin D levels may decline as the disease progresses, making it challenging to determine whether the differences in vitamin D levels are causally linked to the disease course or represent a consequence of the deficiency. It is important to note that the timing of blood sample collection for measuring serum levels of 25-hydroxyvitamin D (25(OH)D) can significantly influence outcomes. Despite these limitations, the observed trends suggest a potential correlation between low serum levels of 25(OH)D and severe COVID-19 symptoms. Nevertheless, despite these promising findings, further research investigating the level of vitamin D and its supplementation is needed.

## 5. Conclusions

There is growing evidence supporting the immune-boosting and anti-inflammatory properties of vitamin D. Pregnant women with COVID-19 often experience vitamin D deficiency, and the level of this vitamin has been shown to have a strong correlation with the severity of the disease. Given the correlation between vitamin D serum levels and COVID-19 symptoms, as well as the occurrence of the infection, it is advisable to consider appropriate vitamin D supplementation during pregnancy.

## Figures and Tables

**Table 1:** The characteristic of studies included in the meta-analysis.

Study	Study Design	Pregnant Women [*n*]	Mean Vitamin D Serum Levels [ng/mL]	Age, Mean [Year]	Outcome	NOS Score
Moreno-Fernandez et al., 2022 [49]	Case control	63 COVID-19	21.28 ± 9.5	31.9	There is no significant difference between pregnant women with COVID-19 and healthy pregnant women.	8
		61 healthy	18.54 ± 8.0	31.5		
Ferrer-Sánchez et al., 2022 [50]	Case control	82 COVID-19	10.15 ± 7.0	31	Vitamin D serum levels were significantly lower in COVID-19. There was no significant difference between intensive care unit vs. non-intensive care unit due to the course of the disease.	8
		174 healthy	13.8 ± 8.5	32		
		75 mild COVID-19	10.5 ± 7.26	NA		
		7 moderate and severe COVID-19	8.7 ± 2.15	NA		
		78 COVID-19 non-intensive care unit	10.15 ± 7.1	NA		
		4 COVID-19 intensive care unit	9.3 ± 4.24	NA		
Schmitt et al., 2022 [51]	Retrospective cohort	15 COVID-19	10.4 ± 9.1	30	Significantly decreased vitamin D serum levels were found in COVID-19-infected and symptomatic pregnant women.	6
		19 healthy	19.02 ± 6.2	31		
		7 asymptomatic COVID-19	13.04 ± 7.95	30.7		
		8 symptomatic COVID-19	10.35 ± 6.12	29.5		
Vásquez-Procopio et al., 2022 [52]	Case control	79 healthy	26.18 ± 3.9	29	Significantly decreased vitamin D serum levels were found in COVID-19 pregnant women.	8
		32 asymptomatic COVID-19	24.98 ± 3.74	30.8		
		44 mild COVID-19	20.75 ± 2.88	30.3		
		10 severe COVID-19	17.1 ± 4.10	31.5		
Seven et al., 2022 [53]	Cross-sectional	292 mild COVID-19	15.5 ± 7.6	28	Vitamin D serum levels were substantially higher in pregnant women with moderate COVID-19 than in those with severe COVID-19.	8
		111 severe COVID-19	13.0 ± 8.9	29.5		
Sinaci et al., 2021 [54]	Case control	159 confirmed COVID-19	12.46 ± 6.46	29.6	Vitamin D serum levels were significantly lower in COVID-19 pregnant women and substantially higher in mild pregnant women relative to moderate and severe COVID-19.	7
		332 healthy	18.76 ± 13.74	27.4		
		128 mild COVID-19	13.69 ± 9.72	NA		
		31 moderate and severe COVID-19	9.1 ± 8.8	NA		
Tekin et al., 2021 [55]	Prospective case control	147 confirmed COVID-19	14.64 ± 10.72	27.9	Significantly increased vitamin D serum levels were observed in COVID-19.	7
		300 healthy	12.52 ± 8.28	27.9		

## Data Availability

Not applicable.

## Supplementary Materials

The following supporting information can be downloaded at: https://www.mdpi.com/article/10.3390/nu15112588/s1, Table S1: PRISMA checklist.
